# Pleuro-Pulmonary Nocardiosis as Opportunistic Infection in a Patient with Chronic Hepatitis C under Combination Treatment with Pegylated Interferon, Ribavirin, and Boceprevir

**DOI:** 10.1155/2013/529041

**Published:** 2013-07-09

**Authors:** Csilla Putz-Bankuti, Harald H. Kessler, Thomas Valentin, Eva Leitner, Emina Talakic, Helmut Schoellnast, Peter Fickert, Guenter J. Krejs, Rudolf E. Stauber

**Affiliations:** ^1^Department of Internal Medicine, Division of Gastroenterology and Hepatology, Medical University of Graz, Austria; ^2^Department of Internal Medicine, LKH Hoergas-Enzenbach, Austria; ^3^Research Unit Molecular Diagnostics and Molecular Diagnostics Laboratory, IHMEM, Medical University of Graz, Austria; ^4^Department of Internal Medicine, Division of Pulmonology, Medical University of Graz, Austria; ^5^Section of Infectious Diseases, Bacteriology and Mycology Laboratory, IHMEM, Medical University of Graz, Austria; ^6^Department of Radiology, Medical University of Graz, Austria

## Abstract

Nocardiosis is an infrequent but serious pulmonary infection caused by Gram-positive aerobic actinomycetes. In this paper, we report on a 48-year-old patient with pleuropulmonary nocardiosis and cirrhosis due to chronic hepatitis C virus infection treated with triple antiviral treatment complicated by prolonged neutropenia.

## 1. Introduction

Pleuropulmonary nocardiosis is an uncommon infection and may cause radiologic findings that vary from vague pulmonary infiltrates to cavitary lesions. It is found more commonly in patients with underlying lung disease and in immunocompromised patients. We describe the first case of this atypical bacterial infection in a patient with hepatitis C virus related liver cirrhosis undergoing antiviral therapy with pegylated interferon alpha, ribavirin, and boceprevir (Merck Sharp & Dome Ltd, Hertforshire, United Kingdom) complicated by prolonged neutropenia.

## 2. Case Report

A 48-year-old Caucasian male was admitted to our inpatient liver clinic in October 2012. He was in poor general condition with ascites, fever (39.4°C), and a dry cough.

In 1997, the patient had been diagnosed with chronic hepatitis C (CHC) subtype 1b which was unresponsive to a 3-month course of interferon (IFN)-alpha monotherapy. In June 2007, he had been admitted to the internal medicine department because of portal decompensation following a period of higher alcohol consumption. CT scan at that time revealed, besides signs of cirrhosis, a right-sided nodular pulmonary lesion measuring 3 × 2 × 2 cm, which was interpreted as posttuberculosis infiltration without structural changes of the bronchial architecture. Following abstinence from alcohol, liver function had stabilized from August 2008 onwards. Antiviral combination treatment with pegylated interferon (pegIFN) alpha-2a 180 *μ*g given weekly and ribavirin (RBV) (600 mg/12 h) had been started in September 2009. Due to the rapid virological response with no detectable hepatitis C virus (HCV) RNA determined with the COBAS AmpliPrep/COBAS TaqMan HCV Test (lower limit of detection 15 IU/mL) at week 4 of treatment, the antiviral therapy had been shortened to 24 weeks. Three months after the end of treatment, the patient developed a virological relapse. In May 2012, the patient had been evaluated for triple antiviral combination treated with the protease inhibitor boceprevir. In June 2012, a 4-week lead-in phase with pegIFN alpha-2a 180 *μ*g given weekly and ribavirin (600 mg/12 h) was started. At week 4, the pegIFN dosage was reduced to 135 *μ*g weekly due to neutropenia and boceprevir (800 mg/8 h) being added. The duration of neutropenia was 3 months with an average absolute neutrophil count of 500/*μ*L. At week 8, HCV RNA was undetectable in serum.

**Figure 1:**
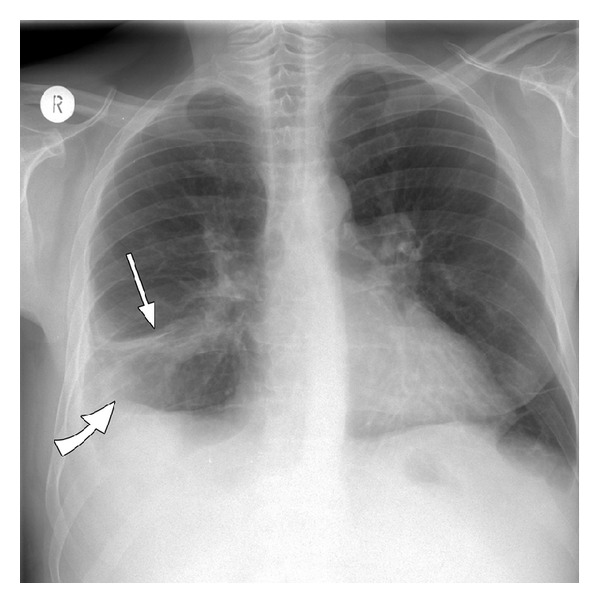
Chest X-ray showing an area of consolidation in the right lower field (straight arrow) with pleural effusion (bent arrow).

**Figure 2:**
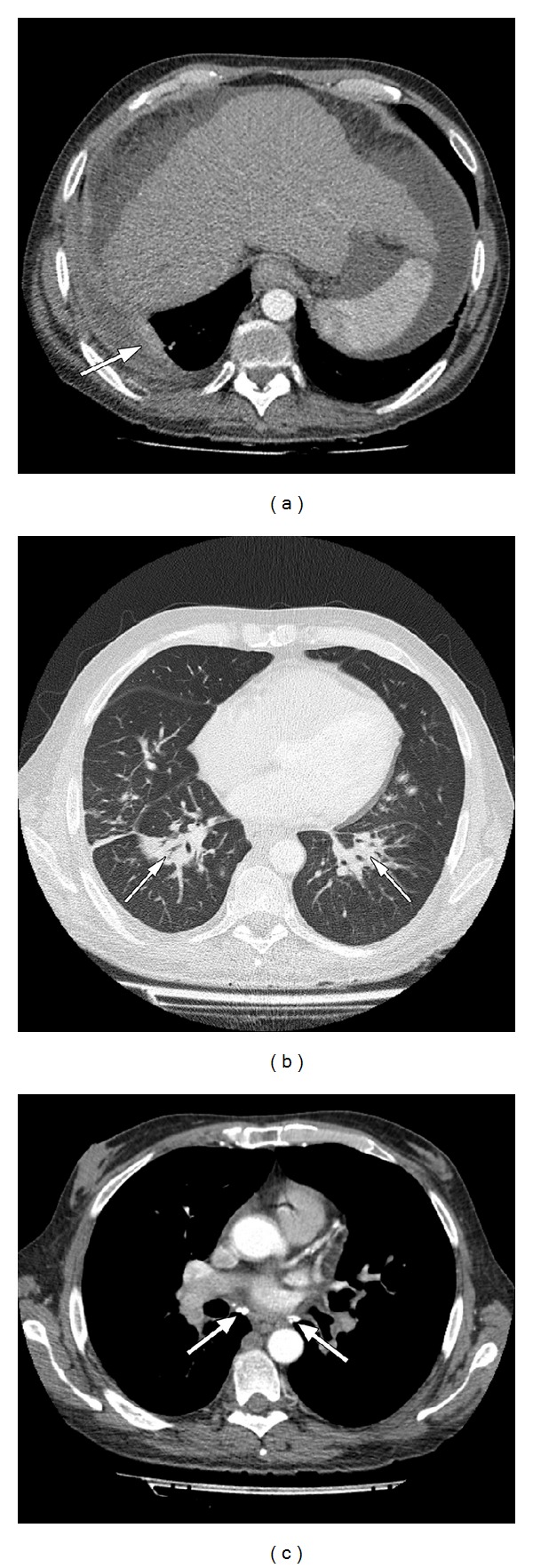
Contrast enhanced helical computer tomography of the thorax. (a) Air space consolidation in both lower lobes (arrows); (b) pleural effusion on the right side with adjacent atelectasis (arrow); (c) calcified bihilar lymph nodes (arrows).

In October 2012 (week 16 treatment), he developed continuous fever. After admission to the inpatient liver clinic, anti-HCV treatment was stopped immediately. Laboratory tests showed leucopenia (white blood cells 2460/*μ*L and neutrophils 1700/*μ*L) and an elevated C-reactive protein of 127 mg/dL (0–5 mg/dL), elevated alanine aminotransferase of 106 U/L (0–50 U/L), and an alkaline phosphatase 159 U/L (35–130 U/L). Reactivation of tuberculosis was unlikely considered due to a negative intradermal tuberculin reaction, negative interferon-gamma release assay, and the absence of acid-fast bacilli in sputum. Chest radiography revealed an area of opacification in the right lower lobe and a pleural effusion on the right side (Figure 1). Thoracic computed tomography confirmed the findings in the chest radiograph and additionally showed consolidation in the left lower lobe and bihilar calcified lymph nodes. No structural changes of the bronchial architecture were seen (Figure 2).

**Figure 3:**
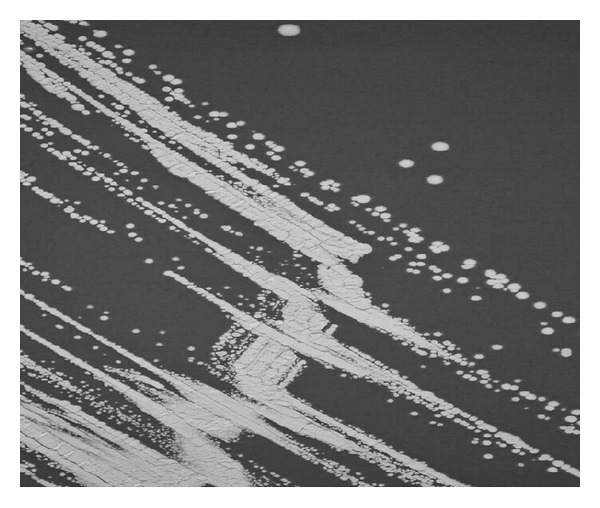
Culture of pleural effusion shows growth of *Nocardia* colonies on chocolate blood agar after 72 h incubation at 35°C.

Cultures of the pleural effusion showed the growth of white colonies on sheep blood agar and chocolate blood agar after a 48 h incubation at 35°C (Figure 3). Gram staining revealed branching filamentous Gram-positive bacilli. For further identification, 16S rRNA gene sequencing was carried out using universal eubacterial primers. Subsequently, a BLAST search of the 1500 bp 16S rRNA gene sequence was performed with the basic local alignment search tool of the National Center of Biotechnology Information (http://blast.ncbi.nlm.nih.gov/Blast.cgi). A homology of 99% was achieved with various *Nocardia *spp. including *Nocardia aobensis*, *Nocardia africana*, *Nocardia veterana*, and *Nocardia elegans.* For antimicrobial susceptibility testing, plates were incubated at 35°C in ambient air, and minimal inhibitory concentrations (MICs) were determined by Etest (bioMérieux, Marcy l'Etoile, France) using an inoculum corresponding to McFarland standard 1.0 on Mueller-Hinton agar plates with 5% sheep blood. MICs obtained after 48 h were amikacin 0.125 *μ*g/mL (susceptible), ceftriaxone 8 *μ*g/mL (susceptible), imipenem 0.125 *μ*g/mL (susceptible), linezolid 2 *μ*g/mL (susceptible), doxycycline 32 *μ*g/mL (resistant), and trimethoprim-sulfamethoxazole 0.064 *μ*g/mL (susceptible).

The patient received trimethoprim-sulfametrole three times daily intravenously (equivalent to 15 mg trimethoprim/kg body weight/day), and a chest drain was placed. After 6 weeks of therapy, the patient showed complete resolution of symptoms, disappearance of the radiological abnormalities, and improvement of liver function. Three months after stopping the antiviral therapy, HCV RNA was 67 IU/mL (limit of detection < 15 IU/mL) corresponding to a virological relapse. 

## 3. Discussion

HCV is a leading cause of chronic liver disease and cirrhosis [1, 2]. Patients suffering from chronic hepatitis C (CHC) are usually immunocompetent but not able to eliminate HCV. Therapy of CHC has recently been improved through addition of direct-acting antiviral drugs such as boceprevir and telaprevir to the standard therapy consisting of pegIFN and RBV. Boceprevir is a NS3/4A protease inhibitor. The duration of boceprevir treatment depends on the virological response 8 weeks after commencing treatment [3, 4].

 This report describes an atypical case of nocardiosis without structural modifications of the bronchial architecture in a patient with CHC and liver cirrhosis under antiviral combination therapy with pegIFN, RBV, and boceprevir. *Nocardia*, a Gram-positive bacterium, is a member of the order *Actinomycetales*. Nocardiosis represents a rare disease mainly affecting individuals with cellular immunodeficiency. This opportunistic pathogen induces disseminated infections with pulmonary, abdominal, bone marrow, and skin manifestations [5–9]. Patients with nocardiosis may present symptoms indistinguishable from those in patients with pulmonary infections of other etiologies. Furthermore, nocardiosis may be difficult to diagnose because of difficulties of identification in culture material. Due to the poor prognosis with a mortality of 15% in cases with delayed diagnosis, an early start of effective therapy is urgently required. *Nocardia* infections were recently observed in CHC liver transplant patients under immunosuppressive regimens [8, 9]. Therefore, it is tempting to speculate that in this case antiviral treatment-induced neutropenia may have favored the manifestation of *Nocardia* infection. In this context, it should be pointed out that in the RESPOND-2 clinical trial the addition of boceprevir to pegIFN/RBV increased the incidence of grade 3 neutropenia significantly (19 and 20% in boceprevir arms versus 9% with standard care) [3]. 

This paper is the first report on pleuro-pulmonary nocardiosis in a patient with HCV-related cirrhosis under triple antiviral treatment including boceprevir complicated by prolonged neutropenia. In conclusion, opportunistic pathogens including *Nocardia* spp. should be considered for the differential diagnosis of pleuro-pulmonary infections of patients with prolonged neutropenia due to severe CHC treatment.
